# Circular RNA HIPK3: A Key Circular RNA in a Variety of Human Cancers

**DOI:** 10.3389/fonc.2020.00773

**Published:** 2020-05-15

**Authors:** Jingyuan Wen, Jingyu Liao, Junnan Liang, Xiao-ping Chen, Bixiang Zhang, Liang Chu

**Affiliations:** Hepatic Surgery Center, Tongji Medical College, Tongji Hospital, Huazhong University of Science and Technology, Wuhan, China

**Keywords:** circular RNA, cancer, circHIPK3, mRNA splicing, biomarker, miRNA sponge

## Abstract

Circular RNAs (circRNAs), which act as initiators and promoters of various diseases, were thought to be mostly noncoding RNAs (ncRNAs) in eukaryotes, until recent studies confirmed that some circRNAs have the function of encoding proteins. Accumulating research findings have proved that dysregulation of circRNAs is associated with the developmental process of multiple cancers. circHIPK3, an example of circRNA, is frequently expressed in many diseases, such as diabetes, age-related cataract, idiopathic pulmonary fibrosis, preeclampsia, osteoblasts, and retinal vascular dysfunction, leading to disease development and progression. In addition, circHIPK3 may also serve as a potential biomarker, to help us know more about the rules of occurrence and development of cancers. In recent studies, many circHIPK3-related cancers have been identified, including nasopharyngeal carcinoma, gallbladder cancer, lung cancer, hepatocellular carcinoma, osteosarcoma, glioma, colorectal cancer, ovarian cancer, bladder cancer, prostate cancer, gastric cancer, oral squamous cell carcinoma, and chronic myeloid leukemia. This review summarizes recent studies on the biological mechanisms of circHIPK3 and expounds the molecular mechanisms of circHIPK3 in these malignant tumors.

## Introduction

The proportion of protein-coding genes in the human genome is ~2%. Non-protein-coding sequences of the human genome account for over 70% of the noncoding RNAs (ncRNAs) (1–3). Recent studies have shown that ncRNAs act as a necessary element in many kinds of biological processes, including post-transcriptional regulation, epigenetics, chromatin modification, and the cell cycle (4, 5). It is increasingly being reported that ncRNAs also affect human cancers and disease progression (6). ncRNAs are subdivided into small (≤ 200 nucleotides) and long (> 200 nucleotides) (7, 8).

**Figure 1:**
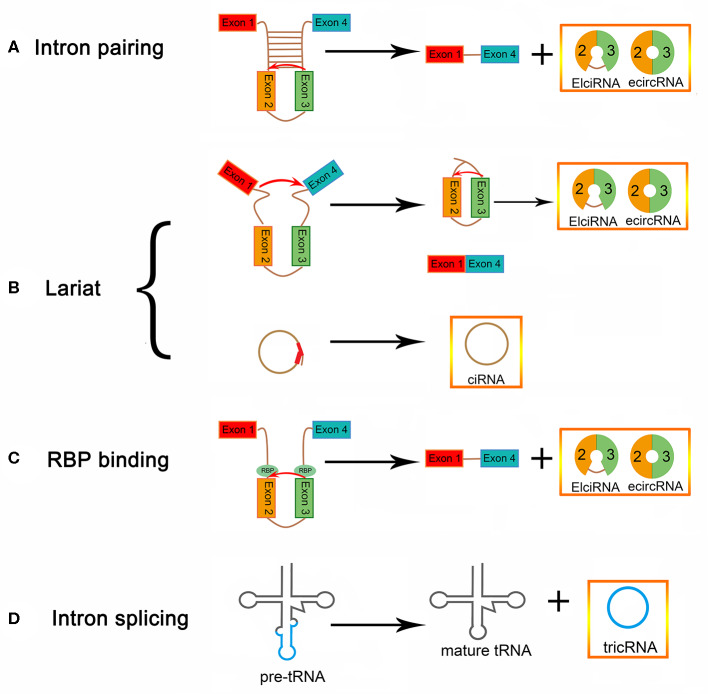
circRNA biogenesis **(A)** EIciRNA biogenesis and ecircRNA biogenesis via intron pairing-driven circularization. **(B)** EIciRNA, ecircRNA, and ciRNA biogenesis via lariat-driven circularization. **(C)** EIciRNA biogenesis via RBP-associated pairing-driven circularization. **(D)** TricRNA biogenesis via intron splicing from pre-tRNA.

The lengths of circular RNAs (circRNAs) are ~200–2,000 bp, with most being around 500 bp. circRNAs are a class of endogenous RNAs that are transcribed by RNA polymerase II (9). They are divided into noncoding and coding circRNAs. Coding circRNAs were found to have an open reading box, which generally contains at least one internal ribosome entry site (10). The closed loop structure can be formed by a back-splicing mechanism, in which an upstream 5′ splice site is linked with a downstream 3' splice site, in a linear mRNA; thus, the regulation of circRNA biogenesis may be influenced by canonical spliceosomal signals (11–14) (Figure 1). circRNAs can be produced from many genomic positions, such as exons, 3′ UTRs, 5′ UTRs, antisense RNAs, intergenic regions, and intronic regions (15, 16). Exonic circRNAs (ecircRNAs), which make up 80% of identified circRNAs, are mainly derived from one or more exons (14, 17, 18). Lariat-driven circularization is one proposed hypothesis for the formation of ecircRNA. The precursor mRNA (pre-mRNA) forms a lariat intermediate, composed of numerous exons and introns (19, 20). The introns are excised, and then the closed loop structure is generated through the “head-to-tail” joining of the upstream 3′ splice site to the downstream 5′ splice site (21, 22). The generation of exon-intron circRNAs (EIciRNAs) occurs, in some cases, when the introns between exons are not spliced out completely (21, 23, 24). Another mechanism of formation of circRNA is intron pairing-driven circularization, which can form a circular structure via the base-pairing of introns flanking inverted repeats. Transfer RNA (tRNA) intronic circular RNAs (tricRNAs) can be generated after removing the excised tRNA introns from tRNA precursors by splicing enzymes and ligating the intron termini (25–27). In addition, RNA-binding proteins (RBPs) cause a difference in circRNA biogenesis, by acting as trans-factors (28). Circular intronic RNAs (ciRNAs) depend on conserved sequences at both ends of the intron and are generated from intron lariats. These conserved sequences can help introns avoid branching, and form circular structures (29).

Multiple studies have revealed that RNA interference could target circRNAs; thus, circRNAs might guide the specific regulation of miRNA-targeted genes by acting as competitive endogenous RNAs (ceRNAs) and miRNA sponges (30, 31). As shown in previous studies, some ecircRNAs are distributed in the cytoplasm and combine with miRNAs to behave as miRNA sponges, to control their function, and the others can interfere with the RNA replication of viruses and viroids (32). However, ciRNAs and EIciRNAs located in the nucleus cannot act as miRNA sponges but can influence gene expression and transcription, by such as cis- or trans-activation (33). Thus, circRNA may regulate protein production and function by binding, storing, sorting, and sequestering proteins to appropriate subcellular locations and may modulate protein-protein interactions (34). In addition, circRNAs can bind to RNA-binding proteins (RBP), in competition with their parent mRNAs, to affect the translation process. Many recent studies have used RNA-seq to analyze the biological functions of circRNAs and have identified various circRNAs, in many model organisms, with different cell types (35, 36). RNase R is the exonuclease of circRNAs, but circRNAs lacking 3′ termini cannot usually be degraded by RNase R, and possess greater stability than the related linear mRNAs. Thus, the concentration of circRNAs in quiescent and postmitotic cells is higher than that of linear mRNAs (37). In addition, genome-wide analyses suggest that circRNAs are abundant in human body fluids, with a highly conserved evolution, and the expression of cell-type-specific circRNAs indicates that they may be regarded as potential biomarkers for predicting disease progression and prognosis (38, 39).

**Figure 2:**
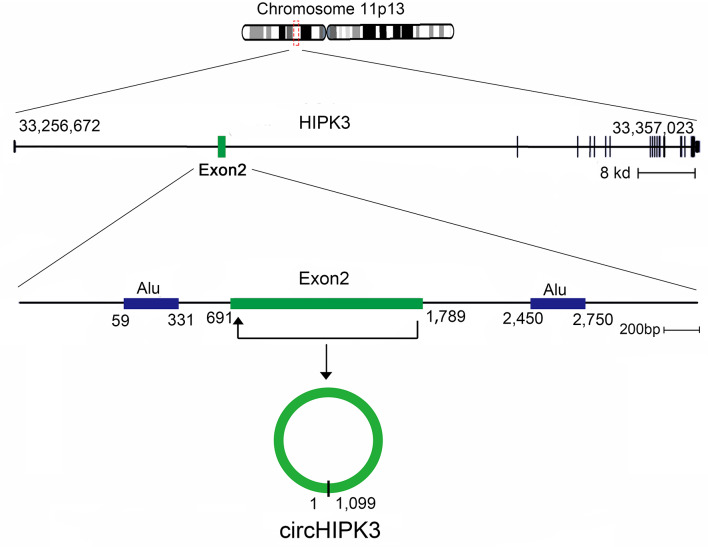
Generation of circHIPK3. *HIPK3* is located on chromosome 11p13, and the genomic regions of *HIPK3* exon 2 contain flanking Alu repeats and long introns. *HIPK3* yields an abundant 1,099 nt circular RNA comprised solely of exon 2.

In this review, we characterize one abundant circRNA, produced from exon 2 of the homeodomain-interacting protein kinase 3 (*HIPK3*) gene, circHIPK3, which has been reported in many recent studies (40). *HIPK3*, Gen Bank Accession ID NM_005734.5, is located on chromosome 11p13 and comprises 7,551 base pairs (Figure 2). The genome sequence indicates that the second exon (1,099 bp) from *HIPK3*, plus the long introns at both ends of the gene combine to make the structure of circHIPK3 (41). circHIPK3 is the dominant circRNA isoform, as evidenced by a mass of back-spliced unique reads (14, 41). The formation of circHIPK3 requires long flanking introns with complementary Alu repeats (19, 42). circHIPK3 is mostly located in the cytoplasm and is commonly expressed in diverse tissues, such as the lung, heart, stomach, colon, and brain (41). Several biological and pathological processes, beyond cancer, are related to the dysregulated expression of circHIPK3. This review summarizes the aberrant expression and molecular mechanism of circHIPK3 in human cancers, and addresses its clinical significance.

## The Function of circHIPK3 in Cancers

### Lung Cancer

**Figure 3:**
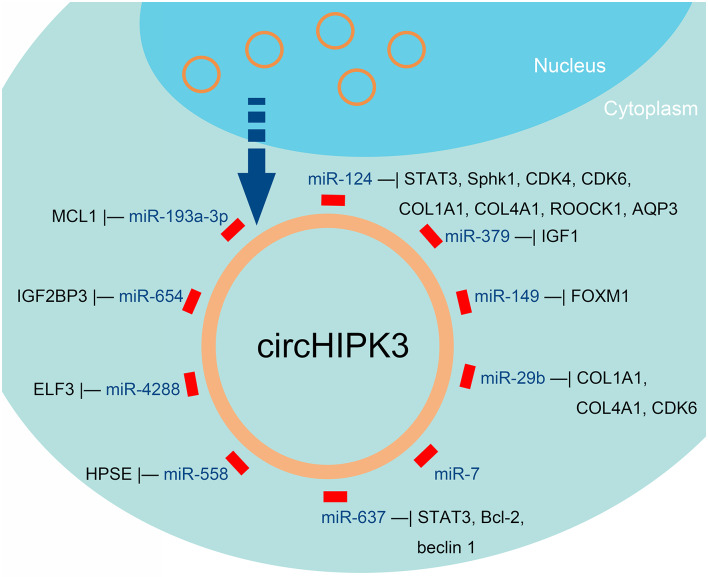
miRNAs that can be sponged by circHIPK3, according to published reports about human cancers.

According to the morphology of tumor cells, lung cancer is classified into two categories: small cell lung cancer (SCLC) and non-SCLC (NSCLC). Chen et al. noted that circHIPK3 is an important autophagy regulator in STK11 mutant lung cancer, by regulating miR-124-3p expression (Figure 3, Table 1) (44). Knockdown of circHIPK3 significantly induced cell autophagy, while cell proliferation, migration, and invasion were impaired. In addition, transfection with an miR-124-3p mimic or abrogation of *STAT3* also induced autophagy. They demonstrated that *STAT3* is the downstream target of miR-124-3p, which influences cell autophagy via the PRKAA/AMPKa pathway. The researchers observed that a high ratio of circHIPK3 to linHIPK3 was a sign of poor prognosis, especially in patients with advanced NSCLC (44). The proliferation of NSCLC cells was investigated by Tian et al. (45). Significantly, overexpression of circHIPK3 accelerated the proliferation of NSCLC cells, and interference of circHIPK3 inhibited it. Moreover, miR-379 can be directly combined with circHIPK3 and IGF1 mRNA. Overexpression of circHIPK3 increased the expression of IGF1, and downregulation of IGF1 was observed with interference of circHIPK3 (45). Furthermore, miR-379 mimics restored the upregulated expression and cell proliferation phenotype induced by IGF1, in a cell line stably expressing circHIPK3. Thus, circHIPK3 increased the expression of IGF1 by reducing miR-379, to accelerate NSCLC cell proliferation (45). In addition to these findings, Lu et al. indicated that miR-149 expression was increased by circHIPK3 silencing. Viability and proliferation of NSCLC cells decreased with miR-149 overexpression. The function of NSCLC cells was regulated by miR-149-mediated FOXM1 expression (46). In the study of Yu et al., circHIPK3 overexpression promoted proliferation in lung cancer cell line A549. The researchers reported that the expression level of circHIPK3 was higher in human lung cancer cells and tissues than in normal tissues. They showed that miR-124 was accumulated upon silencing circHIPK3, induced tumor cell apoptosis, and alleviated tumor cell proliferation. Further studies in tumor tissues demonstrated that downstream target genes (Sphk1, STAT3, and CDK4) were upregulated, suggesting the possibility of identifying therapeutic targets from this axis (43).

**Table 1:** Functional characteristics of circHIPK3 in multiple human cancers.

**Cancer types**	**Expression**	**Roles**	**Functional roles**	**Related genes and protein**	**References**
Lung cancer	Upregulated	Tumor promoter	Viability, proliferation, apoptosis	SphK1, STAT3, CDK4	(43)
	Upregulated	Tumor promoter	Proliferation, migration, autophagy	STAT3	(44)
	N/A	Tumor promoter	Proliferation	IGF1	(45)
	Upregulated	Tumor promoter	Proliferation, migration, invasion, apoptosis	FOXM1	(46)
Gastric cancer	Upregulated	Tumor promoter	Proliferation	COL1A1, COL4A1, CDK6	(47)
	Downregulated	Tumor suppressor	N/A	N/A	(48)
	Upregulated	Tumor promoter	Proliferation, migration	WNT1, TCF4, β-catenin	(49)
Colorectal cancer	Upregulated	Tumor promoter	Proliferation, migration, invasion, apoptosis, metastasis	c-Myb, FAK, IGF1R, EGFR, YY1	(50)
	Upregulated	Tumor promoter	oxaliplatin resistance, autophagy	STAT3, Bcl-2, beclin1	(51)
Bladder cancer	Downregulated	Tumor suppressor	Migration, invasion, angiogenesis, metastasis	HPSE	(52)
Nasopharyngeal carcinoma	Upregulated	Tumor promoter	Proliferation, migration, invasion	ELF3	(53)
Gallbladder cancer	Upregulated	Tumor promoter	Viability, proliferation, apoptosis	ROCK1, CDK6	(54)
Hepatocellular carcinoma	Upregulated	Tumor promoter	Proliferation, migration	AQP3	(55)
Osteosarcoma	Downregulated	Tumor suppressor	Proliferation, migration, invasion	N/A	(56)
Glioma	Upregulated	Tumor promoter	Proliferation, migration, invasion	IGF2BP3	(57)
Epithelial ovarian cancer	Upregulated	Tumor promoter	N/A	N/A	(58)
Prostate cancer	Upregulated	Tumor promoter	Proliferation, invasion	MCL1	(59)
Oral squamous cell carcinoma	Upregulated	Tumor promoter	Proliferation	N/A	(60)
Chronic myeloid leukemia	Upregulated	Tumor promoter	N/A	N/A	(61)

### Gastric Cancer

The function of circHIPK3 in gastric cancer (GC) was investigated by Cheng et al. (47). The level of circHIPK3 was significantly higher in GC tissues than in paired, adjacent normal tissues. In addition, the expression of circHIPK3 in infiltrative-type GC cells was higher than that in expanding-type GC cells, suggesting that circHIPK3 expression is related to aggressive clinical factors in GC. GC cell proliferation was inhibited by circHIPK3 knockdown and promoted by circHIPK3 overexpression. The researchers performed bioinformatic analyses and identified *COL1A1, COL4A1*, and *CDK6* as common targets in the circHIPK3-miR-124/miR-29b axes (47). Ghasemi et al. investigated the clinical significance of circHIPK3 (48). Its expression was downregulated in GC tumoral tissues and was significantly correlated with various clinicopathological parameters. However, Liu et al. found that circHIPK3 levels were higher in GC tissues than in paracancerous tissues (49). Similarly, its level was higher in GC cells than in normal cells. In addition, an inverse correlation between circHIPK3 expression level and overall survival of GC patients was found. Silencing of circHIPK3 weakened the proliferative and migratory abilities of GC cells (49). Additionally, circHIPK3 knockdown markedly downregulated the levels of WNT1, TCF4, and β-catenin (49). circHIPK3 promoted tumor cell proliferation and migration through the Wnt/β-catenin signaling pathway, which is a classic proliferation-related pathway (62). Their results also showed that upregulation of circHIPK3 indicated poor prognosis in patients with GC (49).

### Colorectal Cancer

As a malignancy of the digestive tract, colorectal cancer (CRC) is a widespread disease with high incidence and fatality rate and is a significant threat to human health (63). Experimental induction of highly expressed circHIPK3 *in vivo* and *in vitro* was first carried out in 2018 (50). These data indicated that the c-Myb/circHIPK3/miR-7 axis might be a therapeutic target against CRC. The results proved that circHIPK3 expression in CRC was significantly higher than in normal tissues and that increased circHIPK3 expression indicates poor prognosis. Downregulation of circHIPK3 suppressed cell invasion, proliferation, and migration, and induced apoptosis in CRC cells. Moreover, c-Myb, an upstream transcription factor, elevated circHIPK3 expression by directly binding to its promoter region. Furthermore, circHIPK3 can sponge miR-7 in CRC cell lines and upregulate the expression of oncogenes (*FAK, IGF1R, EGFR*, and *YY1*) to promote CRC progression (50). Recent studies have found that the main cause of recurrence in CRC patients is resistance to oxaliplatin-based chemotherapy (51, 64). Aiming at the miR-637/STAT3/Bcl-2/beclin1 axis, circHIPK3 in CRC cells functioned as a chemoresistant gene. Therefore, circHIPK3 might be used as a prognostic prediction for oxaliplatin-based chemotherapy in CRC patients (51). The transcriptomic data from the Gene Expression Omnibus database (GSE116589) revealed that circHIPK3 is highly expressed in CRC tissues and exosomes from CRC patients (65, 66).

### Bladder Cancer

circHIPK3, also known as bladder cancer-related circular RNA-2 (BCRC-2), was found to be downregulated in bladder cancer and negatively correlated with cancer grade, invasion, and lymph node metastasis (52, 67). circHIPK3 overexpression efficiently inhibited cell migration, invasion, and angiogenesis *in vitro*, while cell growth and metastasis were suppressed *in vivo*. miR-558 can bind with circHIPK3 at two critical sites and is sponged abundantly to suppress heparanase (HPSE) expression (52).

### Nasopharyngeal Cancer

Ke et al. demonstrated that the expression of circHIPK3 was higher in nasopharyngeal cancer (NPC) tissues and cells, a common malignant tumor occurring in the head and neck region, than in normal tissues and cells (53). circHIPK3 overexpression enhanced tumor cell proliferation and increased colony numbers, and circHIPK3 silencing dramatically inhibited the migration and invasion of NPC cells, suggesting that circHIPK3 exerts an oncogenic role. The effect of miR-4288 on the expression of E74-like ETS transcription factor 3 (ELF3) was counteracted by overexpression of circHIPK3 overexpression, but not by mutant circHIPK3. In addition, the level of ELF3 in NPC tissues increased with an increase in circHIPK3 expression. This study demonstrated that circHIPK3 upregulated ELF3 expression by reducing the levels of miR-4288, and the regulatory circHIPK3-miR-4288-ELF3 axis plays a critical role in NPC progression (53).

### Gallbladder Cancer

Kai et al. showed that the level of circHIPK3 in human gallbladder cancer (GBC) was higher than that in gallbladder epithelial cells (54). Cell apoptosis was induced when circHIPK3 silencing, by siRNA, downscaled the survival and proliferation of GBC cells. They found that the levels of miR-124 targets (ROCK1 and CDK6) increased upon circHIPK3 inhibition of the activity of miR-124 in GBC cells. Moreover, the upregulation of ROCK1-CDK6 and downregulation of miR-124 were correlated with circHIPK3 in GBC tissues (54). Notably, CDK4 and CDK6 are involved in the Ras/Raf/MAPK pathway, which transfer signals from the extracellular environment to the nucleus. This signaling pathway plays a key role in the occurrence and development of malignant tumors, such as by stimulating angiogenesis via changing the expression of genes involved in neovascularization, cell cycle regulation, integrin signaling, and cell migration (68). Hence, circHIPK3 may affect the progression of GBC through the Ras/Raf/MAPK pathway (54).

### Hepatocellular Carcinoma

Hepatocellular carcinoma (HCC) is a fatal carcinoma, with nearly 6 × 10^5^ deaths each year (69). Quantitative reverse transcription-polymerase chain reaction (qRT-PCR) analyses validated that the expression of miR-124 was decreased in HCC tissues, whereas adjacent nontumor liver tissues had normal miR-124 expression (55). Chen et al. (55) demonstrated that miR-124 inhibited the proliferation and migration of HCC cells. In addition, the 3′ UTR of aquaporin 3 (*AQP3*) contained an miR-124 binding motif. Elevating the expression of AQP3 promoted tumor proliferation and migration. circHIPK3 knockdown decreased HCC cell proliferation and migration rate, which was rescued by an miR-124 inhibitor. Also, inhibition of miR-124 overcame the inhibition of *AQP3* by circHIPK3. circHIPK3 knockdown suppressed Huh7 xenograft tumor growth and results indicated that circHIPK3 sponged miR-124 to regulate AQP3 expression in HCC (55).

### Osteosarcoma

Studies of circHIPK3 in bone cancers have focused on osteosarcoma (OS), which is more common in children and adolescents (70). Ma et al. (56) found that circHIPK3 expression was downregulated in OS tissues and plasma samples, which is associated with poor prognosis. Functional analysis revealed that, in OS cells, proliferation, migration, and invasion were significantly suppressed when circHIPK3 was overexpressed *in vitro* (56).

### Glioma

Glioma originates from the neuroepithelium of the brain, accounting for the majority of intracranial tumors (71). Jin et al. found that circHIPK3 was upregulated in glioma tissues, and higher levels of circHIPK3 were associated with worse prognosis. A series of experiments showed that circHIPK3 accelerated tumor cell proliferation, invasion, and metastasis *in vivo*. Moreover, circHIPK3 promoted IGF2BP3 expression by targeting miR-654 in glioma cells. Overexpression of IGF2BP3 reversed the effects of circHIPK3 depletion (57).

### Ovarian Cancer

Ovarian cancer (OC) is a type of gynecological malignancy that causes massive mortality worldwide, and the pathology of most OC is epithelial ovarian cancer (EOC) (72). It was demonstrated using qRT-PCR analysis that the expression level of circHIPK3 is significantly increased in EOC tissue samples over matched, adjacent noncancerous tissues (58). The experimental results indicated that higher lymph invasion rates and shorter survival times were positively correlated with circHIPK3 expression in EOC patients (58).

### Prostate Cancer

Chen et al. indicated that circHIPK3 overexpression was associated with prostate cancer (PCa) malignancy and poor prognosis (59). They noted that downregulation of circHIPK3 significantly inhibited malignant phenotypes, including proliferation, migration, and invasion *in vitro*, and tumor growth *in vivo*. They found that miR-193a-3p functioned as a tumor suppressor in PCa cells, through bioinformatic prediction and biotin-coupled miRNA capture studies. Functional studies showed that miR-193a-3p reduced MCL1 expression, to suppress proliferation, migration, and invasion in cancer cells (59).

### Oral Squamous Cell Carcinoma

Wang et al. explored the expression and clinical applications of circHIPK3 in oral squamous cell carcinoma (OSCC) and the effect of miR-124 expression, regulated by circHIPK3, on OSCC cell proliferation (60). The circHIPK3 expression in OSCC tissues was significantly higher than that in paracancerous tissues. The expression of circHIPK3 in cancer tissues at different clinical stages and with varying degrees of cell differentiation was observed to be statistically different. The proliferation ability of OSCC cells was significantly inhibited by reducing the expression of circHIPK3. Moreover, miR-124 expression in OSCC tissues was significantly lower than that in paracancerous tissues. There was a negative correlation between circHIPK3 and miR-124 expression in OSCC tissues. Taken together, downregulating the expression level of circHIPK3, which is upregulated in OSCC, inhibited the proliferation of OSCC cells, and circHIPK3 contributed to the occurrence and development of OSCC by regulating the expression of miR-124 (60).

### Chronic Myeloid Leukemia

Feng et al. investigated the expression of circHIPK3 in chronic myeloid leukemia (CML). They found that circHIPK3 was significantly upregulated in peripheral blood mononuclear cells and serum samples from patients with CML compared with those from healthy controls. High circHIPK3 expression predicted poor outcomes in patients with CML. Further loss-of-function experiments suggested an oncogenic role of circHIPK3 in CML (61).

## The Function of circHIPK3 in Cancers

### Clinical Features of circHIPK3

As mentioned above, circHIPK3 is able to block circularization, like other circRNAs, which allows it to escape the actions of exonucleases. Therefore, circHIPK3 can exist stably in cells for a long time (6). It is precisely because of its stability that we can easily determine whether circHIPK3 can be a potential biomarker molecule for clinical diagnosis (Table 2).

**Table 2:** Clinical features associated with circHIPK3 in multiple human cancers.

**Cancer types**	**Clinicopathological feature**	**References**
Gastric cancer	Poorer overall survival, Higher in infiltrative type GC cell, Advanced TNM stage	(47)
	Age, M classification	(48)
	Poor prognosis	(49)
Colorectal cancer	Advanced TNM stage, Lymph node metastasis, Distant metastasis, Advanced tumor	(50)
	Tumor size, Regional lymph node metastasis, Distant metastasis, Survival	(51)
Bladder cancer	Advanced tumor, Lymph node metastasis, Grade high	(52)
Nasopharyngeal carcinoma	Poorer overall survival, Lower distant metastasis-free survival rate, Advanced tumor	(53)
Hepatocellular carcinoma	Tumor differentiation, Advanced TNM stage, HBV-DNA copy numbers, Liver cirrhosis	(55)
Osteosarcoma	Enneking stage, Lung Metastasis, Poor prognosis	(56)
Glioma	Poorer overall survival	(57)
Epithelial ovarian cancer	Lymph node invasion, Advanced FIGO stage	(58)
Prostate cancer	Advanced tumor	(59)

circHIPK3 has been shown to be closely related to multiple clinical features, through the study of bioinformatics, statistics, and various types of cancer patient. In solid tumors, high expression of circHIPK3 often represents larger tumor mass and volume. More advanced tumor morphology is often found in cancer tissues that highly expressed circHIPK3. Distant metastasis and lymph node metastasis often represent poor prognosis, which is usually observed in patients with high circHIPK3 expression, suggesting that reduced overall survival is related to circHIPK3 in gastric cancer, colorectal cancer, bladder cancer, nasopharyngeal carcinoma, hepatocellular carcinoma, osteosarcoma, glioma, epithelial ovarian cancer, prostate cancer, and so on. At the same time, increased differentiation and advanced TNM stages are associated with abnormal expression of circHIPK3. In addition, Zhang et al. also noted a relationship between circHIPK3 and resistance to chemotherapy drugs, such as the relationship between oxaliplatin resistance and circHIPK3, thus establishing a causative link. These findings suggest that circHIPK3 is a promising target in cancer therapy (51).

## The Function of circHIPK3 in Other Diseases

The function of circHIPK3 has also been reported in many other diseases. circHIPK3's role in diabetes was the first to be investigated. Stoll et al. suggested that circHIPK3 is a novel regulator of β-cell activities, and that β-cell dysfunction is an essential hallmark of diabetic conditions (73). They showed that circHIPK3 acts by sequestering miR-124-3p and miR-338-3p to regulate the expression of crucial target genes, including *Slc2a2, Akt1*, and *Mtpn*. Cao et al. showed that the circHIPK3-miR-124 pathway participated in high glucose (HG)-induced endothelial cell injury (74). circHIPK3 was downregulated by the accumulation of miR-124 in both HG-treated human umbilical vein endothelial cells and primary aortic endothelial cells, from diabetic patients. According to research on type 2 diabetes patients, by Wang et al., a positive association was shown between circHIPK3 expression and the grade of neuropathic pain (75). They demonstrated that neuropathic pain could be alleviated in diabetic rats when circHIPK3 was silenced. Further mechanistic studies illustrated that circHIPK3 negatively regulated the expression of miR-124. As additional proof, in diabetic rats with circHIPK3 knockdown, inhibition of miR-124 would mediate the moderation of neuropathic pain, thereby inhibiting neuroinflammation (75).

George et al. (76) suggested that circHIPK3 may regulate human lens epithelial cell (HLEC) function through miR-193a-mediated crystallin alpha A (CRYAA) expression. They found that circHIPK3 was significantly downregulated in all subtypes of age-related cataract. The silencing of circHIPK3 significantly accelerated apoptosis upon oxidative stress and decreased cell viability and proliferation (76). Meanwhile, circHIPK3 knockdown inhibited epithelial-mesenchymal transition and increased miR-193a expression. miR-193a reduced the mRNA and protein levels of CRYAA by targeting the binding sites in its 3′ UTR. Furthermore, miR-193a decreased the viability, promoted proliferation, and increased the apoptosis of HLECs under conditions of oxidative stress (77).

Zhang et al. illustrated that circHIPK3 silencing could improve idiopathic pulmonary fibrosis and inhibit fibroblasts (78). Substantial evidence was found that circHIPK3 increases SOX4 and COL1A1 expression by inhibiting miR-338-3p activity, as an endogenous sponge. Furthermore, these results revealed that, *in vitro*, circHIPK3 plays a critical role in the emergence of TGF-β-induced myofibroblasts, predominantly through the fibroblast-to-myofibroblast transition (FMT). From this research, the correlation between the circHIPK3-miR-338-3p/SOX4/COL1A1 network and FMT regulation was revealed (78).

Zhang et al. found that circHIPK3 was decreased in patients with preeclampsia and affected biological processes such as the migration and invasion of human trophoblast cells (79). The levels of circHIPK3 detected in preeclampsia were significantly lower than those in the normal pregnancy group. Silencing circHIPK3 inhibited cell migration, invasion, proliferation, and tubular formation, but circHIPK3 overexpression effectively promoted these abilities in addition to promoting cell proliferation (79).

Dexamethasone (DEX) exerts cytotoxicity to osteoblasts by inducing apoptosis and necrosis. Zhu et al. found that circHIPK3 expression was downregulated in DEX-treated human osteoblasts and necrotic femoral head tissues (80). Overexpression of circHIPK3 suppressed DEX-induced apoptosis and programmed necrosis, and knockdown of circHIPK3 enhanced DEX-induced cytotoxicity in osteoblasts. They found that miR-124 could induce cytotoxicity by DEX in human osteoblasts. Conversely, the DEX-induced actions were attenuated when miR-124 was sponged by circHIPK3 (80).

## Conclusions and Prospects for Future Research

Several studies have validated that circRNAs regulate gene expression and are closely related to tumorigenesis and tumor progression, in a complicated gene regulatory network. In this review, we provide an overview of many new characterizations of circHIPK3 that may deepen our understanding of how it regulates the process of tumor development. In addition, the relationship between circHIPK3 and clinical features was also examined. Various experiments have demonstrated that the expression of circHIPK3 might be a marker of accelerated tumor progression, both *in vitro* and *in vivo*. circHIPK3 expression was related to increased tumor volume of tumors, advanced TNM stage, shortened survival, and metastasis. Meanwhile, circHIPK3 has potent effects in controlling the cell cycle, influencing cell proliferation, regulating apoptosis, promoting invasion, and managing migration in cancers. A new target could be provided by circHIPK3 for diagnosing and treating different types of cancer. Moreover, a growing number of studies have demonstrated the mechanisms of circHIPK3 in several pathways, such as the Wnt/β-catenin signaling pathway. It is well known that there is competition between circHIPK3 and miRNAs, which results in the inhibition of the downstream genes of those miRNAs.

However, findings on the role of circHIPK3 are limited; further experiments are needed to explore the function of circHIPK3 and its pathway, in a range of human malignancies. Due to technical limitations, research has focused on the role of circHIPK3 as a miRNA sponge, and its other functions need further study. In addition, the mechanism of circHIPK3 in other diseases may provide new ideas and considerations for future research on malignant cancers. In addition to *C-MYB*, there may be other significant genes regulating the expression of circHIPK3, which represents a future direction of study. It is worth noting that, because of the complex mechanisms associated with circHIPK3, it may play an opposite role in different cancers, and within the same tumor, with circHIPK3 promoting the development of some tumors, while inhibiting the proliferation and growth

## Author Contributions

JW and JLiao collected the related paper. JW, JLiao, JLian, and LC drafted and revised the manuscript. JW and LC designed the review. BZ and XC participated in the design of the review and helped to draft and revise the manuscript. All authors read and approved the final manuscript.

## Conflict of Interest

The authors declare that the research was conducted in the absence of any commercial or financial relationships that could be construed as a potential conflict of interest.

## Abbreviations

ncRNAs: Noncoding RNAs
circRNAs: circular RNAs
ecirRNAs: exonic circRNAs
ElciRNAs: exon-intron circRNAs
tRNA: transfer RNA
tricRNAs: tRNA intronic circRNAs
RBPs: RNA-binding proteins
ciRNAs: circular intronic RNAs
ceRNA: Competitive endogenous RNAs
HIPK3: Homeodomain-interacting protein kinase3
NSCLL: Non-small-cell lung cancer
SCLL: Small-cell-lung cancer
STAT: Signal transducer and activator of transcription 3
PRKAA: Protein kinase AMP-activated catalytic subunit alpha 2
AMPKa: AMP-activated protein kinase alpha subunit
IGF1: Insulin like growth factor 1
FOXM1: Forkhead box M1
SPHK1: Sphingosine kinase 1
CDK4: Cyclin-dependent kinase4
GC: gastric cancer
COL1A1: collagen type I alpha 1 chain
COL4A1: collagen type IV alpha 1 chain
CDK6: Cyclin-dependent kinase6
WNT1: Wnt family member 1
TCF4: Transcription factor 4
CRC: Colorectal cancer
c-Myb: MYB proto-oncogene
FAK: Focal adhesion kinase
IGF1R: Insulin like growth factor 1 receptor
YY1: YY1 transcription factor
Bcl-2: B cell leukemia/lymphoma 2
GEO: Gene Expression Omnibus
BCRC-2: Bladder cancer-related circular RNA-2
HPSE: Heparinase
BC: Bladder cancer
NPC: Nasopharyngeal carcinoma
ELF3: E74-like ETS transcription factor 3
GBC: Gallbladder cancer
ROCK1: Rho associated coiled-coil containing protein kinase 1
MAPK: Mitogen activated kinase-like protein
HCC: Hepatocellular carcinoma
qRT-PCR: quantitative reverse transcription polymerase chain reaction
AQP3: 3′-UTR of aquaporin3
OS: Osteosarcoma
IGF2BP3: Insulin like growth factor 2 mRNA binding protein 3
CCK8: Cell counting kit-8
OC: Ovarian cancer
EOC: Epithelial ovarian cancer
Pca: Prostate cancer
MCL1: Myeloid cell leukemia sequence 1
OSCC: Oral squamous cell carcinoma
CML: Chronic myeloid leukemia
SLc2a2: Solute carrier family 2 member 2
AKT1: AKT serine/threonine kinase 1
Mtpn: Myotrophin
HG: High glucose
HLEC: Human lens epithelial cell
CRYAA: Crystallin alpha A
3′-UTR: 3′-untranslated region
SOX4: SRY-box transcription factor 4
TGF-β: Transforming growth factor-β
FMT: Fibroblast-to-myofibroblast transition
DEX: Dexamethasone
OS: Overall Survival.
